# Impact of medical school experiences on the career choice of neurosurgery: a cross- sectional study from Pakistan

**DOI:** 10.1186/s12909-024-05452-9

**Published:** 2024-04-26

**Authors:** Muhammad Shakir, Hammad Atif Irshad, Eisha Abid Ali, Amna Adil, Ahmed Altaf, Syed Ather Enam

**Affiliations:** 1 Department of Neurosurgery, Aga Khan University Hospital, Karachi, 74800 Pakistan; 2 Medical College, Aga Khan University, Karachi, 74800 Pakistan; 3 University College of Medicine and Dentistry, Lahore, Pakistan; 4 Rawalpindi Medical University, Rawalpindi, Pakistan; 5 Section of Neurosurgery, Department of Surgery, Aga Khan University, Stadium Road, Karachi, 74800 Pakistan

**Keywords:** Clinical rotations, Career choice, Neurosurgery, Medical education

## Abstract

**Background:**

Pakistan grapples with the issue of an inadequate neurosurgery workforce but the reasons for such a disparity remain uncertain. Previous studies have highlighted how various factors including medical school experiences have an impact on career choice, but no study has delved into the impact of medical school exposure among Pakistani medical students, especially for the field of neurosurgery. This study aims to evaluate the impact of neurosurgery exposure, mentorship, and interest groups on medical students' decision to pursue neurosurgery.

**Methods:**

A national cross-sectional survey was conducted in Pakistan, collecting data from medical students, interns, and medical officers over a one-month period. Ethical approval was obtained from the Ethical Review Committee at Aga Khan University, Pakistan. The data was analyzed using SPSS version 26.

**Results:**

Out of 2618 participants, 38.0% were male and 62.0% were female, with an average age of 21.82 years (± 2.65). Among them, only 358 (13.6%) were interested in pursuing neurosurgery as a career, while the remaining 2,260 (86.3%) were not. More females (58.9%) than males (41.1%) expressed interest in pursuing neurosurgery as their intended career. Most medical students interested in pursuing neurosurgery were in the early years of their medical school (1st Year: 19.6%, 2nd Year: 26.0%, 3rd Year: 20.9%). In our study, students from public sector institutions (52.2%) showed more interest in neurosurgery as a career choice compared to those from private sector institutions (44.1%). The main deterrents for choosing neurosurgery were intense training (42.2%), work-life balance (39.9%), limited residency slots (56.7%), medical knowledge (34.1%), and surgical skills (36.6%).

**Conclusion:**

This study highlights the need for increased student engagement to inculcate the decision to pursue neurosurgery among medical students in Pakistan. A significant gap is highlighted, with the majority of interested students in early years. Public sector students show higher interest than their private sector counterparts. However, barriers like intense training, limited residency slots, and work-life balance concerns influence career choice. Targeted interventions like mentorship programs are crucial for fostering future neurosurgeons and advancing patient care and research. By addressing the identified disparities in experiences and promoting a supportive educational environment, it is possible to cultivate a future generation of skilled and dedicated neurosurgeons who can contribute to advancements in patient care and research in the field.

## Introduction

The global need for neurosurgical care is expanding and according to the World Federation of Neurosurgical Societies (WFNS), a minimum neurosurgery workforce density should be 1 per 200,000 population for optimum access to neurosurgical care. Pakistan faces a significant shortage of neurosurgeons, with only an estimated 212, reported in 2016 by WFNS [1]. This glaring deficit in the field contributes to the growing burden of neurosurgical care. The global increase in numbers compared to the decline in our charts is alarming and could stem due to multiple factors. These could be attributed to the lack of exposure, limited presence of neurosurgeon mentors, coupled with a shortage of comprehensive training courses, limited residency slots and the omission of it from the curriculum in pre-clinal years, collectively lead to failure in attracting and providing quality experiences to medical students [2, 3].

In Pakistan, medical students enter the clinical care arena from the third year of medical school [4]. Through this clinical training phase, also known as clerkship, develop initial impressions regarding various specialties. These impressions are based on experiences they have had and are linked to hands-on experience, patient interaction, effective mentorship, positive opinion of teacher role models, approachable staff, salary structure, and general perception in the community [5]. Students rotate across various specialties but very few institutions offer rotation for subspecialties, especially surgical specialties like neurosurgery. Consequently, missing out on exposure to numerous specialties that they could have a potential interest in [6].

Human beings carry a wealth of memories, emotions, and associations learned from their past encounters [7]. These experiences serve as a foundation for interpreting, evaluating potential risks and making future judgments [8]. In context to this, studies from across the globe have highlighted that medical students who had positive experience in a particular field during undergraduate rotations ended up pursuing it, suggesting that experiential influences play a crucial role in shaping specialty decisions [5, 9–12]. Therefore, the objective of our study is to provide an outlook on the student perspective of selecting neurosurgery as a career in Pakistan. The aim of this study is to assess whether exposure to neurosurgery, meaningful mentorship, interest groups or university societies plays a role in influencing students to pursue a career in neurosurgery.

## Materials and methods

A cross-sectional survey was conducted among medical students, interns, and medical officers in Pakistan over a one-month period. Ethical approval was obtained from the Ethical Review Committee (ID: 2023–8484-24,189) at the Aga Khan University, Pakistan. The study targeted current medical students and recent graduates from both private and public sectors in Pakistan.

### Sample size and population

To determine the sample size, previous literature suggested comprising of medical students was considered [13]. Using the OpenEpi website, a minimum sample size of 383 was calculated with a 5% precision value and 95% confidence interval [14].

### Eligibility criteria

The survey targets current medical students and recent graduates from both private and public sectors in Pakistan. Inclusion criteria include individuals currently enrolled in Pakistani medical schools or those who have graduated within the last five years. Participants may belong to private or public medical institutions and must voluntarily consent to participate in the study. Current medical students must be enrolled in any year of their medical program, while recent graduates must have completed their medical degree within the specified timeframe. Exclusion criteria include individuals not meeting the specified enrollment or graduation criteria, and those who do not consent to participate. Additionally, participants previously involved in similar surveys within the past year are excluded.

### Data collection tool and coding

Data was collected using a self-designed online questionnaire, formulated by the research team in collaboration with faculty specializing in student surveys and neurosurgery research at the Aga Khan University. The questionnaire covered demographics, institute details, neurosurgery opportunities, perceptions, barriers, career choices, and reasons for pursuing a career in neurosurgery. A pilot study with 50 medical students was conducted to validate the questionnaire, and necessary modifications were made based on their feedback.

### Sampling technique

As a national survey in Pakistan, there were no official platforms for medical students to access the survey. To address this, an ambassadorship program was created by the research team, ensuring equal distribution of data collectors from all provinces. Various methods, including online platforms, face-to-face interactions, and dissemination within surgery and neurosurgery interest groups in medical colleges, were used for data collection.

### Statistical analysis

Data analysis was performed using IBM Statistical Package for Social Sciences (SPSS) version 26. Descriptive statistics were used to report demographic characteristics, with normally distributed continuous data presented as mean ± standard deviation and categorical data presented as frequencies and percentages (n; %). Chi-squared tests were used to analyze survey responses and identify any significant differences between subgroups. A *p*-value < 0.05 was considered significant.

## Results

Out of the 2,618 participants in our study, 358 individuals (13.6%) had neurosurgery as their choice of career, while 2,260 participants (86.3%) did not. More females answered neurosurgery as their intended career, with 58.9% of female participants expressing interest compared to 41.1% of males. A greater proportion of intention to pursue was found in earlier medical school years. Specifically, the percentages of interest were 19.6% in the 1st year, 26.0% in the 2nd year, and 20.9% in the 3rd year. Students from public sector institutions displayed a higher level of interest in career choice of neurosurgery, with 52.2% expressing intention to pursue as a career, compared to 44.1% of students from private sector institutions. Demographic characteristics are outlined in Table 1.

**Table 1:** Demographic characteristics

Variable	Career Choice *N* = 358	Not the Choice of Career *N* = 2260	Total *N* = 2618	*P*-value
	**n (%)/ X̄ ± SD**	**n (%)/ X̄ ± SD**	**n (%)/ X̄ ± SD**	
**Age (years)**	21.21** ± 2.22**	21.91** ± 2.7**	21.82** ± 2.65**	
**Gender**				**0.194**
Male	147(41.1)	847(37.5)	994(38.0)	
Female	211(58.9)	1413(62.5)	1624(62.0)	
**Year of Medical School**				**0.000**
1st Year	70(19.6)	253(11.2)	323(12.3)	
2nd Year	93(26.0)	455(20.1)	548(20.9)	
3rd Year	75(20.9)	547(24.2)	622(23.7)	
4th Year	36(10.1)	251(11.1)	287(11.0)	
5th Year	55(15.4)	417(18.4)	472(18.0)	
Intern	8(2.2)	163(7.2)	171(6.5)	
Medical Officer	9(2.5)	85(3.8)	94(3.6)	
Recent Graduate/Transition Year	12(3.4)	90(4.0)	102(3.9)	
**Province**				**0.000**
Azad Jammu and Kashmir	6(1.7)	29(1.3)	35(1.3)	
Balochistan	6(1.7)	37(1.6)	43(1.6)	
Gilgit Baltistan	0(0)	2(0.1)	2(0.1)	
Islamabad	21(5.8)	117(26)	156(6)	
Khyber Pakhtunkwa	45(12.6)	185(8.2)	230(8.5)	
Punjab	172(48)	1382 (58.7)	1500(55.2)	
Sindh	108(30.2)	545(24.1)	653(24)	
**Sector**				0.028
Public	187(52.2)	1021(45.2)	1208(46.1)	
Private	158(44.1)	1116(49.4)	1274(48.6)	
Semi-Private	13(3.6)	124(5.5)	137(5.2)	
**Monthly Household Income in PKR**				**0.899**
< 100,000	117(32.7)	743(33.2)	860(33.1)	
100,000–150,000	72(20.1)	462(20.6)	534(20.6)	
150,000–200,000	54(15.1)	283(12.6)	337(13.0)	
200,000–300,000	37(10.3)	242(10.8)	279(10.7)	
300,000–400,000	21(5.9)	148(6.6)	169(6.5)	
400,000–500,000	20(5.6)	112(5.0)	132(5.1)	
> 500,000	37(10.3)	250(11.2)	287(11.0)	

The correlation of different sources of exposure to neurosurgery in medical school life with the career choice of medical students is summarized in Table 2. We found that 64.8% of medical students have neurosurgery in their curriculum and among them almost 70% of the medical students are inclined to choose neurosurgery as a future career (*p* = 0.036). Out of 72.7% respondents who agreed to having a medical school/hospital associated neurosurgery department, 73.5% and 72.5% had neurosurgery as their career choice and do not have neurosurgery as their choice of career, respectively. A greater proportion of respondents (53.6%) with a neurosurgery training program in their institute were found in the neurosurgery as a career choice group. The highest (61%) rate of selecting neurosurgery as field of specialization was found among those students who did not rotate in neurosurgery as compared to students (27.9%) who rotated in neurosurgery and this difference was found to be significant (*p* < 0.001).

**Table 2:** Medical school characteristics

Question	Career Choice	Not the Choice of Career	Total	*P*-Value
	**n (%)**	**n (%)**	**n (%)**	
**Does your medical school have a curriculum that includes neurosurgery?**				0.036
No	108(30.3)	812(36.0)	920(35.)	
Yes	249(69.7)	1446(64)	1695(64.)	
**Does your medical school affiliated institute/hospital have a neurosurgery department?**				0.724
No	35(9.8)	253(11.2)	288(11.0)	
Yes	263(73.5)	1639(72.5)	1902(72.7)	
Maybe	60(16.8)	368(16.3)	428(16.3)	
**Is there a neurosurgery training/residency program offered at your institute?**				0.428
No	60(16.8)	372(16.5)	432(16.5)	
Yes	192(53.6)	1142(50.5)	1334(51.0)	
Maybe	106(29.6)	746(33.0)	852(32.5)	
**Have you rotated in neurosurgery?**				0.000
No	218(60.9)	1480(65.5)	1698(64.8)	
Yes (As a Medical student)	100(27.9)	606(26.8)	706(27.0)	
Yes (As an intern/house officer)	9(2.5)	109(4.8)	118(4.5)	
Yes (As a Medical officer)	9(2.5)	23(1.0)	32(1.2)	
Yes (As an Elective/Observer student)	22(6.1)	43(1.9)	65(2.5)	
**Does your institute provide opportunities to take part in neurosurgery research?**				0.088
No	86(24.0)	469(20.7)	555(21.2)	
Yes	157(43.9)	934(41.3)	1091(41.7)	
Not Sure	115(32.1)	858(37.9)	973(37.2)	
**Does your institute have a neuroscience or neurosurgery interest group?**				0.007
No	154(43.0)	870(38.5)	1024(39.1)	
Yes	99(27.7)	535(23.7)	634(24.2)	
Maybe	105(29.3)	856(37.9)	961(36.7)	

**Fig. 1:**
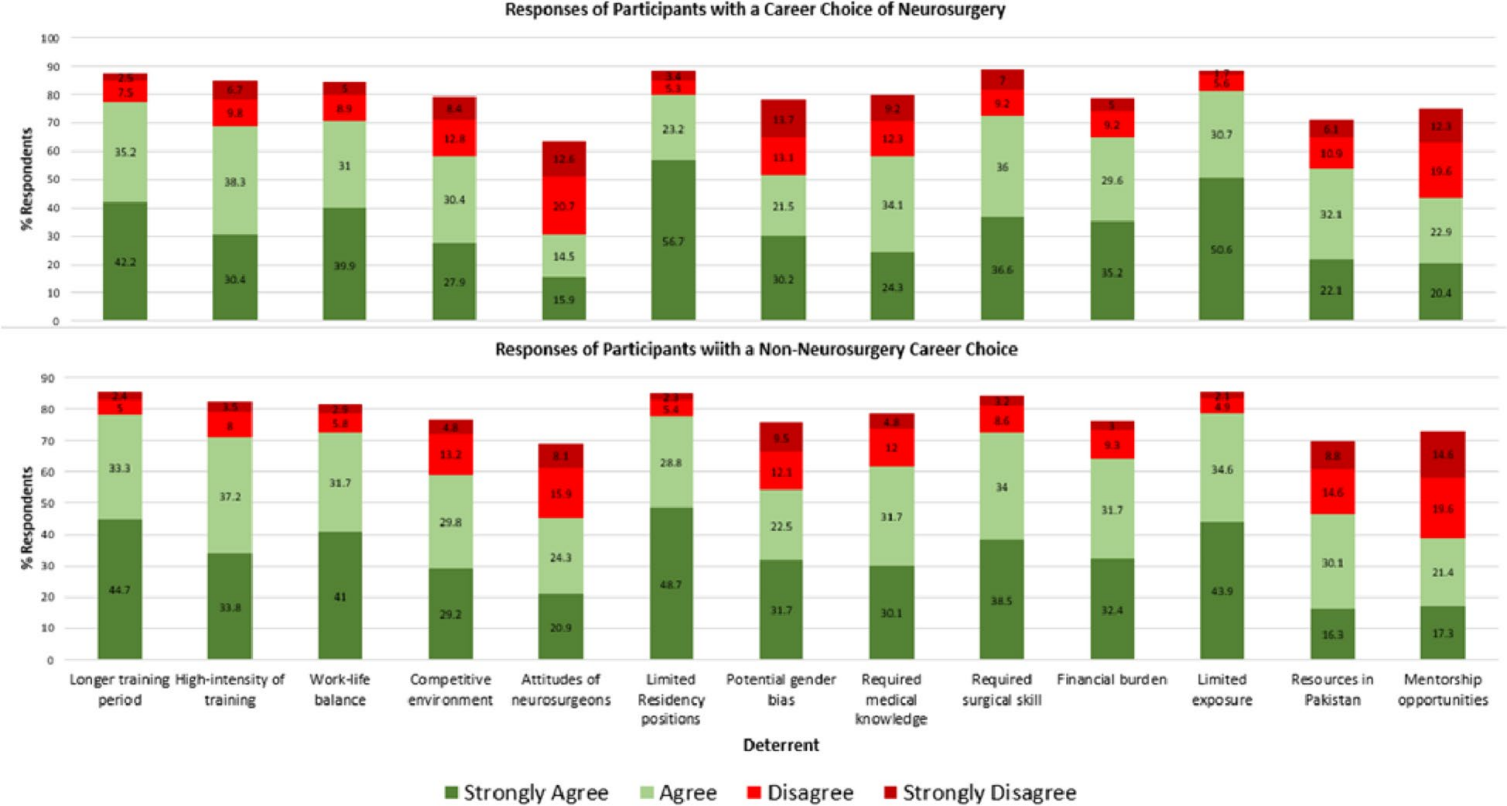
Deterrents to pursuing neurosurgery according to participants

Amongst students opting for neurosurgery as a career, our study identifies deterrents towards neurosurgery. The main deterrents identified were the perceived intensity of training (42.2%), concerns regarding work-life balance (39.9%), limited availability of residency slots (56.7%), perceived gaps in medical knowledge (34.1%), and the need for strong surgical skills (36.6%). As seen in Fig. 1.

## Discussion

Out of 2618 respondents, a remarkable difference of 72.6% was seen among students considering neurosurgery as a choice of career with 358 students considering neurosurgery as a career choice while 2260 students didn't show any interest in pursuing the field.

The objectives of neurosurgery education in medical school are twofold: 1) to give all medical students fundamental concepts and key learning objectives related to neurosurgical disease; and 2) to offer suitable candidates a pathway of exploration, education, and mentorship to pursue further training in neurosurgery. In our study, 60.9% of participants could not get a chance to rotate in neurosurgery clerkship rotation and these were the students who wanted to pursue a career in the field. These factors restrict the students' exposure to the large and fascinating area of neurosurgery and put something of a "ceiling" on their comprehension of the neurosurgical field, which reduces the likelihood that they will choose neurosurgery as a career [15]. Our study revealed a surprising trend: the highest rate of interest in neurosurgery was found among students who had not rotated in the specialty, compared to those who had. This underscores the complex dynamics influencing career decisions. Real-world exposure to neurosurgery may temper idealistic perceptions, highlighting the sacrifices required. However, it is crucial to recognize these challenges as integral to the dedication demanded by the field, rather than deterrents.

We found that the year of study played a significant role with surprisingly, only 2.2% of students in their final year expressing an inclination towards the field, which is noteworthy because the final year of medical school is pivotal for making career choices [16]. The findings of this study are comparable to other studies where 76.96% of students changed their career preferences in their final year of medical school due to factors such as future marital plans, family considerations, early settlement, better income, and improved work conditions [17]. A similar study from the United States that initially proposed medical career plans can be changed due to parental responsibilities and childbearing students particularly among women who do not opt for neurosurgery as a career compared to their non-parent counterparts due to hectic work hours and intense training [17]. This finding warrants further exploration to understand the underlying factors influencing students' career decisions in this field. It is crucial to consider the potential impact of various factors such as personal experiences, exposure to neurosurgery, and the broader socio-cultural context within medical education. While some students may make informed decisions based on realistic exposure to the rigors and demands of neurosurgical practice, others may be dissuaded due to negative experiences, including instances of bullying, discrimination, or a problematic organizational culture.

Another study from Nigeria reported that students from the final year believed that early exposure had the potential to alter their initial decision of not pursuing a career in neurosurgery. It underscores the significance of creating a supportive learning environment and effective teaching methods during neurosurgical clerkships to foster interest [18].

The disparity in career choice preference could shed light on the pre-clinical experience of medical students in the country. Third year is often the start of clinical rotations in Pakistani medical schools and interestingly, we found in our study that among all the medical school years, the third-year cohort exhibited the highest percentage, approximately 24%, of disinterest in opting for neurosurgery as their future career path [19]. A discrepancy in medical student experiences in the field of neurosurgery was seen in our study. Our results showed that a significant number of students, around 60% did not rotate in neurosurgery and notably these were the students who wanted to opt for neurosurgery as a career. Another study from Pakistan highlights the impediments faced during clinical learning in the form of suboptimal clinical rotation planning, insufficient clinical orientation, poor supervision and lack of resources with a crippled feedback delivery system acting as barriers to effective clinical learning. However, due to a lack of adequate clinical rotation planning, students are distanced from several medical fields [20].

Similarly, a study from Saudi Arabia found that interest in neurosurgery as a specialization can be strengthened by enhancing the exposure of students and interns to the specialty during their medical school years. Spending a few days or weeks at the local neurosurgical unit, sitting in clinics, scrubbing in theaters, and joining the daily ward rounds would give students an accurate insight into the specialty and help them grasp what a career in this field might be like [21]. Furthermore, for students hailing from third-world countries, international health electives (IHEs) are significant to incorporate and experience clinically advanced and technologically sophisticated systems available to the first word medical student [22]. However, these results conflicted with the findings of a study which says that an early exposure to the operative room has improved the comprehension of neurosurgery, and it influenced decreasing the interest in the specialty as a career [10]. A study from Johns Hopkins Medical School also documented the impact of an early exposure on enhancing the under representation of medical students in surgery by proposing the introduction of a summer research program exclusively dedicated to facilitating research with robust and focused mentorship, yielding promising results [23].

Many students who rotated in neurosurgery availed the opportunity through electives (6.1%), which provided them with the gateway to customize their own learning experience [24]. This statistic emphasizes the significant potential and enthusiasm of students within this field. According to a study conducted in Pakistan, students believed that electives would help them in their career in terms of getting experience and a chance to explore more. They expected that it would improve their medical knowledge, professionalism, and enhance the quality of care they would be providing [22].

We also found that those who intend on pursuing neurosurgery had greater access to research through their institutions. The results of a study showed that medical students got more insight into neurosurgery specialty after attending the conference related to neurosurgery. Exposure to neurosurgeons through conferences and teaching provided by them positively influences medical students in choosing neurosurgery as a career [25]. Therefore, research is crucial at the undergraduate level and more exposure needs to be incorporated in Pakistani institutions.

Interestingly, students who expressed an interest in neurosurgery were notably absent from any neuroscience interest groups (43.0%). On the other hand, among those who were not interested in neurosurgery, a smaller percentage lacked participation in such groups (38.5%). This suggests that when individuals have a genuine passion for neurosurgery, they tend to proactively seek out opportunities and experiences, even in the absence of formal interest groups or structured support.

Deterrents that are reported in our study are a longer training period, limited exposure, and residency positions. Work life balance was considered the most important factor in discouraging medical students from choosing neurosurgery [26]. In a cross-sectional study at Nepal, it is seen that long training and inadequate facilities for treatment were the few reasons for medical students to become repellent from neurosurgery [27]. Less availability of mentors also played a part in restraining medical students from neurosurgery [28]. The already less available number of neurosurgical residents and consultants in Pakistan leads to production of less mentors to guide the aspiring students about neurosurgery, hence creating a paradoxical cycle [29]. A study by Sandeepa showed that effective leadership and workshops for surgical residents may not only create better future surgeon educators but may also increase the number of students pursuing surgical training [30].

In addition to addressing student motivation, future studies should delve deeper into the systemic challenges limiting the advancement of neurosurgery in Pakistan. Structural deficiencies such as the lack of standardized training programs, insufficient supervision, and the absence of a robust research culture are significant barriers that must be overcome. Furthermore, addressing the financial constraints and inconsistent selection criteria for neurosurgery training is crucial to fostering a conducive environment for aspiring neurosurgeons. By advocating for systemic reforms and proposing practical solutions to these challenges, we can pave the way for a more robust and sustainable neurosurgical workforce in Pakistan.

## Recommendations

Based on our findings we propose the establishment of university-based interest groups to cultivate students' interest, along with increased faculty involvement in leading educational initiatives and collaborative research efforts. Furthermore, the creation of both national and international associations could facilitate early exposure to neurosurgery for medical students. Additionally, modifying the curriculum to enhance its implementation and addressing stigmas associated with pursuing the field could serve as effective initiatives.

## Limitations

The limitation of our study is that our study is a web-based survey, thereby reducing the reliability of responses and interpretation of questions and thus our sample size is not the equal representative of all the provinces of Pakistan. Moreover, results were not reported by institutions, and so some of the institutes might be overrepresented in our sample. Although we sampled 2618 participants, representing approximately 2.5% of the total medical student population in Pakistan, this sample size may not fully capture the diversity of perspectives within the entire population. Being a cross-sectional study, we were also unable to comment on how experiences over the years of medical school impacted the outcome of career choice and therefore our hypothesis requires a follow-up cohort and qualitative study to also incorporate subjective perspectives. Due to the self-reporting nature of the survey, there may be response bias and the possibility of social desirability bias influencing participant responses. Additionally, some medical students may lack clinical exposure to neurosurgery, which could bias their career choice towards or away from neurosurgery based on incomplete information or experiences.

## Conclusion

This study sheds light on the impact of medical experience on the career choice of neurosurgery among medical students and the factors that influence their interest in this field. Based on the findings of our study, it is evident that there are diverse factors influencing medical students' decisions regarding pursuing neurosurgery as a career. While a proportion of student’s express interest in neurosurgery, a significant number do not, citing various deterrents such as the perceived intensity of training, work-life balance concerns, limited residency slots, and challenges related to medical knowledge and surgical skills. Furthermore, our study highlights the need for further exploration into the impact of exposure to neurosurgery during medical education, particularly in evaluating the quality of rotations and understanding students' perceptions and experiences. By addressing these factors and fostering a supportive learning environment, medical educators and policymakers can better equip aspiring neurosurgeons and improve the overall landscape of neurosurgical practice in Pakistan.

## Data Availability

Available from the corresponding author.
